# Molecular Characterization and Functional Analysis of Two Steroidogenic Genes TSPO and SMAD4 in Yellow Catfish

**DOI:** 10.3390/ijms22094505

**Published:** 2021-04-26

**Authors:** Fang Chen, Chong-Chao Zhong, Chang-Chun Song, Shu-Wei Chen, Yang He, Xiao-Ying Tan

**Affiliations:** Key Laboratory of Freshwater Animal Breeding, Ministry of Agriculture, Fishery College, Huazhong Agricultural University, Wuhan 430070, China; chenfang95@webmail.hzau.edu.cn (F.C.); zhongchongchao@webmail.hzau.edu.cn (C.-C.Z.); songchangchun@webmail.hzau.edu.cn (C.-C.S.); chenshuwei@webmail.hzau.edu.cn (S.-W.C.); heyang1997@webmail.hzau.edu.cn (Y.H.)

**Keywords:** *Pelteobagrus fulvidraco*, steroidogenesis-related genes, molecular characterization, promoter, transcriptional regulation

## Abstract

The steroid hormones are required for gonadal development in fish. The present study was undertaken to characterize the cDNA and promoter sequences of TSPO and SMAD4 genes in yellow catfish *Pelteobagrus fulvidraco*, explored the mRNA tissue expression and deciphered their promoter regions. Yellow catfish TSPO and SMAD4 shared the similar domains to the corresponding genes from other vertebrates. The TSPO and SMAD4 mRNAs were widely expressed in the detected tissues, but at different levels. Several transcription factors were predicted, such as Sp, GATA, AP1, SOX1, SRY, STAT, HNF4α, PPARγ, Pu.1 and FOXL2. PPARγ overexpression increased but STAT3 overexpression reduced TSPO promoter activity, and FOXL2 overexpression inhibited the promoter activity of TSPO and SMAD4. The site mutation and EMSA analysis indicated that TSPO promoter possessed STAT3 and FOXL2 sites. Overall, our provided the novel understanding into the transcriptionally regulatory mechanisms of TSPO and SMAD4 in fish.

## 1. Introduction

Steroid hormones modulate embryonic development, sex differentiation, metabolism and reproduction in vertebrates. Translocator protein (TSPO), located in the mitochondrial outer membrane, plays the important roles in the transport of cholesterol to the inner mitochondrial membrane, where cholesterol was converted into pregnenolone by cytochrome P450scc (CYP11A1) [1,2]. Mothers against decapentaplegic homolog 4 (SMAD4) acts as a co-regulator and mediates the transcriptional regulation of CYP19A1 [3], and CYP19A1 is responsible for the formation of C_18_ steroids, which is the most important enzyme in the control of sex development in the fish [4]. Owing to the importance of TSPO and SMAD4 in maintaining the steroidogenesis, it is very essential to study their molecular characterization and the transcriptional regulation. At present, several studies have explored the functional characterizations of TSPO and SMAD4 in several physiological processes [5]. In mammals, the TSPO promoter was cloned and identified from human breast cancer cells, and the binding sites of specificity protein (SP1, SP3 and SP4) positively regulate the activity of TSPO promoter [6]. Similarly, the promoter of SMAD4 was isolated and cloned from patients with thyroid tumor, demonstrating the importance of GC box on the activity of SMAD4 [7]. However, information associated with the molecular characterization and transcriptional regulation of TSPO and SMAD4 promoters were very scarce in fish.

In eukaryotic organisms, promoter regions have many cis-acting elements and regulate the expression of genes at the transcriptional level by binding with transcriptional factors [8]. Therefore, in order to study the regulatory mechanism of TSPO and SMAD4 genes, we should at first explore the structure and function of their promoters. At present, several transcriptional factors, such as Sp sites and activator protein-1 (Ap1), were predicted at TSPO and SMAD4 promoters [9,10]. In addition, signal transducer and activator of transcription 3 (STAT3), peroxisome proliferator-activated receptor gamma (PPARγ) and forkhead box l2 (FOXL2) are three important transcription factors that regulate the expression of many target genes involved in numerous physiological processes, including steroidogenesis [11,12]. Considering that TSPO and SMAD4 are also the key enzymes and transcription factors in regulating steroidogenesis [1,3], we hypothesized that PPARγ, STAT3 and FOXL2 regulated steroidogenesis by targeting TSPO and SMAD4.

Yellow catfish *Pelteobagrus fulvidraco*, widely distributed freshwater fish, is an important economic fish in some Asian countries due to the high economic value and delicious meat quality. At present, studies were scarce on the regulation of steroidogenesis for the fish species [13]. To this end, we cloned and characterized the cDNA and promoter sequences of TSPO and SMAD4 genes, and determined their mRNA tissue expression and investigated their transcriptional regulation in yellow catfish. Our study provides new understanding into characterizing the role of steroid-related genes during ovarian development.

## 2. Results

### 2.1. Molecular Characterization

The full-length cDNAs of TSPO and SMAD4 genes from *P. fulvidraco* were 952 bp and 2221 bp in length, respectively (Table 1). Their predicted amino acid sequences TSPO and SMAD4 were identical to those from other fish and mammals, and the amino acid sequence identities between TSPO and other species ranged from 39.63% to 69.12%, and SMAD4 from 63.93% to 94.19% (Table 2).

The TSPO protein sequence from *P. fulvidraco* had the five α-helix transmembrane structure (TM1–5), the C-terminal cholesterol-recognition amino acid consensus domain (CRAC) (Figure S1). The *P. fulvidraco* SMAD4 possessed the MH1 domain at the N terminus, the MH1 domain at the C terminus, the SMAD4 activation domain (SAD), the DNA binding motif, the nuclear localization and export signals (NLS and NES) (Figure S2).

### 2.2. Tissue Distribution of Gene Expression

**Figure 1:**
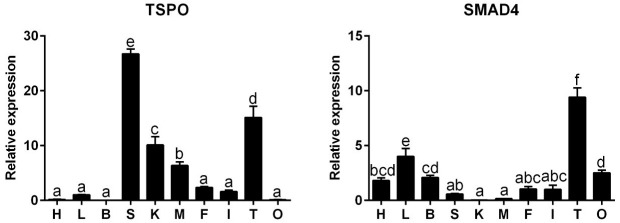
Quantitative PCR (Q-PCR) analysis for mRNA expression of TSPO and SMAD4 across heart (H), liver (L), brain (B), spleen (S), kidney (K), muscle (M), fat (F), intestine (I), testis (T) and ovary (O) of *P. fulvidraco*. Data (mean ± SEM, *n* = 3 replicates. For each replicate, 4 fish were sampled) were expressed relative to expression of reference gene (β-actin and ubce). Bars that share different letters indicate significant differences among various tissues (*p* < 0.05).

TSPO and SMAD4 mRNA expression levels were detected in the tested tissues from *P. fulvidraco*, but their mRNA levels were variable among the tissues (Figure 1). TSPO mRNA levels were predominant in the spleen, followed by the testis, kidney and muscle. SMAD4 mRNA levels were the highest in the testis, followed by the liver and ovary, and the lowest in the kidney.

### 2.3. Sequence Analysis of the Promoter Regions of TSPO and SMAD4

**Figure 2:**
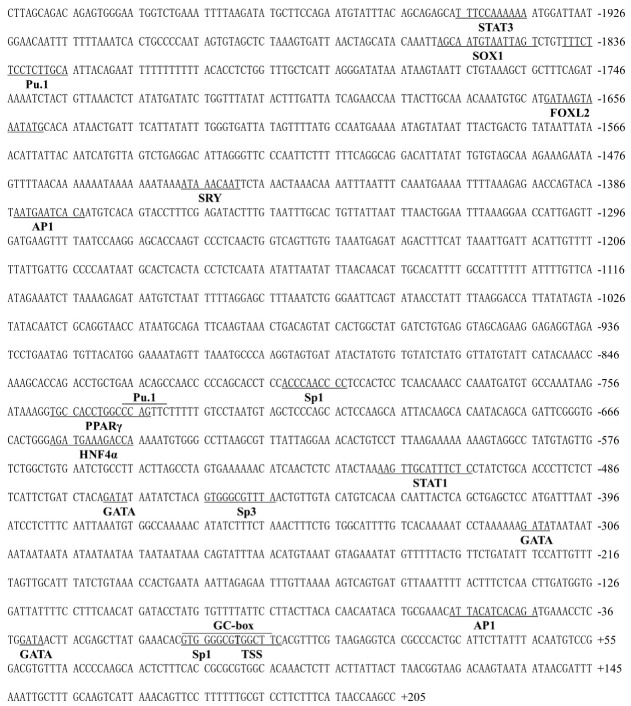
Nucleotide sequences and putative regulatory elements of the TSPO promoter in *P. fulvidraco*. Numbers are relative to the transcription start site (+1). The putative regulatory elements are indicated in bold letters below the underlined sequence.

**Figure 3:**
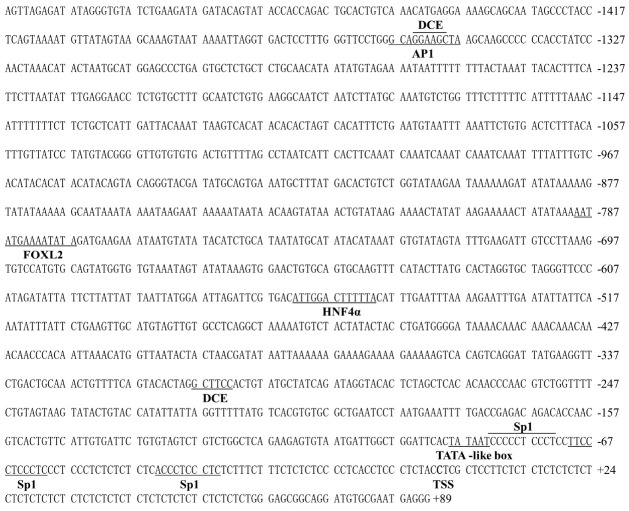
Nucleotide sequences and putative regulatory elements of the SMAD4 promoter in *P. fulvidraco*. Numbers are relative to the transcription start site (+1). The putative regulatory elements are indicated in bold letters below the underlined sequence.

We obtained the 2015 bp of TSPO and the 1506 bp of SMAD4 promoters. On the TSPO promoter region (Figure 2), three binding sites for Sp factors (Sp1/3) were located in position +7 bp to −8 bp, −794 bp to −803 bp, respectively, and −445 bp to −455 bp, respectively, two AP1 binding sites between −44 bp and −55 bp, and −1344 bp and −1384 bp, respectively, and one PPARγ binding site at −734 bp to −748 bp, and a single putative STAT3 site at the position −1936 bp to −1946 bp. Other putative binding sites included FOXL2, GATA, HNF4α, SOX1, SRY, STAT1 and Pu.1 at their promoter regions. On the SMAD4 core promoter (Figure 3), a TATA-like box was located from −83 bp to −88 bp, three Sp1 located from −34 bp to −43 bp, −60 bp to −70 bp and −72 bp to −82 bp, respectively. Other binding sites included DCE, HNF4α, FOXL2 and AP1 on the promoter region of SMAD4 gene.

### 2.4. 5’-Deletion Assay of the Regions of TSPO and SAMD4 Promoters

**Figure 4:**
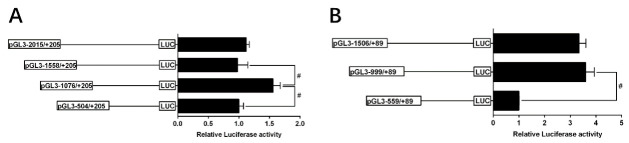
5′ unidirectional deletion analysis of the TSPO and SMAD4 promoters for yellow catfish. Schematic diagram of truncated promoters is shown at left panel. The corresponding luciferase reporter assay results are shown at right panel. A series of plasmids containing 5′ unidirectional deletions of the TSPO (pGL3 −2015/+205, −1558/+205, −1076/+205, −504/+205) and SMAD4 (pGL3 −1506/+89, −999/+89, −559/+89) promoter regions fused in frame to the luciferase gene were transfected into HEK293T cells. Values represent the ratio between firefly and Renilla luciferase activities, normalized to the control plasmid pGl3 −504/+205 (**A**) and pGl3 −559/+89 (**B**), respectively. Results are shown as mean ± standard error of mean (SEM) (*n* = 3). Hash symbol (#) means significant differences between two groups (*p* < 0.05).

The sequence deletion from −1076 bp to −504 bp of TSPO promoter significantly decreased the luciferase activity of the TSPO promoter. Subsequent absence from −1558 bp to −1076 bp significantly increased its luciferase activity and the sequence deletion from −2015 bp to −1558 bp showed no significant effect (Figure 4A). These results demonstrated that the −1076 to −504 bp sequence contained the positively regulatory elements for TSPO expression, and the −1558 to −1076 bp sequence contained the negatively regulatory elements for TSPO expression. For the SMAD4 promoter, the sequence deletion from −999 bp to −559 bp reduced the luciferase activity, whereas the sequence deletion between −1506 bp and −999 bp did not influence its luciferase activity (Figure 4B).

**Figure 5:**
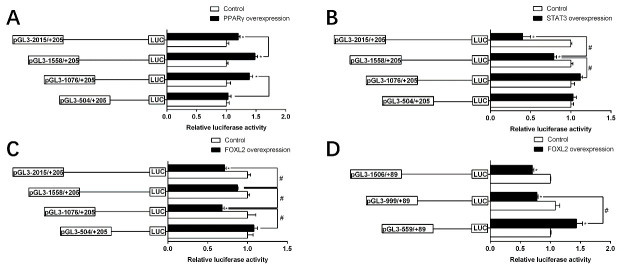
Overexpression analysis of 5′ unidirectional deletion assays of the TSPO and SMAD4 promoters of yellow catfish. (**A**) PPARγ overexpression; (**B**) STAT3 overexpression and (**C**,**D**) FOXL2 overexpression. Values represent the ratio between firefly and Renilla luciferase activities, normalized to the control. Results are shown as mean ± standard error of mean (SEM) (*n* = 3). Hash symbol (#) means significant differences between two groups (*p* < 0.05). Asterisk (*) indicate significant differences between different treatments with the same plasmid (*p* < 0.05).

Since SMAD4 promoter region has no putative PPARγ and STAT3 sites, we next only examined the transcriptional activity of TSPO with 5′-deletion mutants which was influenced by PPARγ and STAT3 overexpression on TSPO. Compared with the control, PPARγ overexpression up-regulated the promoter activity. The deletion from −1076 bp to −504 bp alleviated the PPARγ overexpression-induced TSPO promoter activity, and from −2015 bp to −1558 bp increased PPARγ overexpression-induced TSPO promoter activity, indicating that −1076/−504 and −2015/−1558 regions of TSPO promoter were affected by PPARγ overexpression (Figure 5A). On the contrary, the promoter activity was decreased under the STAT3 over-expression, and the TSPO promoter region from −2015 bp to −1558 bp further enhanced the inhibition of STAT3 overexpression. Nonetheless, the sequence deletion of TSPO promoter between −1076 bp and +205 bp region completely abolished the STAT3-induced down-regulation, reflecting that the negative response elements existed at −2015/−1558 bp and −1558/−1076 bp regions of TSPO promoter to STAT3 (Figure 5B). 

The response of these promoters to FOXL2 overexpression was investigated (Figure 5C,D). For the TSPO promoter, the deletion plasmids of −1076/−504 and −2015/−1558 markedly alleviated the FOXL2 overexpression-induced TSPO promoter activity (Figure 5C). For the SMAD4 promoter, overexpression of FOXL2 markedly reduced the promoter activity compared to the control. The inhibitory effect by FOXL2 was completely abolished when the sequence between −999 and −559 bp was deleted, indicating that there are negative response elements at −999/−559 bp region of SMAD4 promoter to FOXL2 (Figure 5D). 

### 2.5. Site-Mutation Analysis of Binding Sites on the Regions of TSPO and SMAD4 Promoters

**Figure 6:**
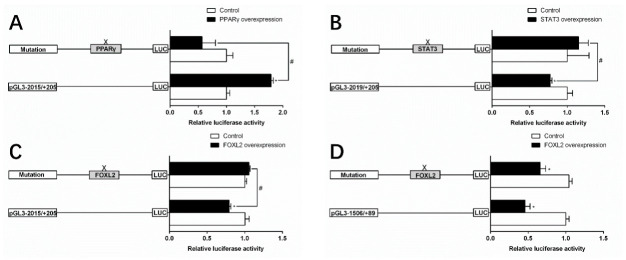
Assays of predicted PPARγ, STAT3 and FOXL2 binding sites after site-directed mutagenesis. (**A**) Site mutagenesis of PPARγ on −2015/+205 TSPO promoter. (**B**) Site mutagenesis of STAT3 on −2015/+205 TSPO promoter. (**C**) Site mutagenesis of FOXL2 on −2015/+205 TSPO promoter. (**D**) Site mutagenesis of FOXL2 on −1506/+89 SMAD4 promoter. Values are presented as mean ± SEM (*n* = 3). Hash symbol (#) means significant differences between two groups (*p* < 0.05). Asterisk (*) indicate significant differences between different treatments with the same plasmid (*p* < 0.05).

Next, we performed the site mutation to further elucidate whether TSPO and SMAD4 promoters possessed functional binding sites of PPARγ, STAT3 and FOXL2 (Figure 6). The mutation of the −734/−748 PPARγ binding site reduced the PPARγ overexpression-induced luciferase activity significantly, demonstrating that this site positively mediated TSPO transcription (Figure 6A). Compared with the pGl3 −2019/+205 TSPO vector, the mutation of STAT3 binding site between the −1507 and −1516 significantly escalted the luciferase activity in the STAT3 overexpression group, indicating the STAT3 site inhibited STAT3 overexpression-induced TSPO transcription (Figure 6B). Overexpressed FOXL2 reduced TSPO promoter activity compared to the control, but the mutated FOXL2 abolished its inhibitory effect, demonstrating that FOXL2 site inhibited FOXL2-induced TSPO transcription (Figure 6C). And the mutation of the SMAD4-FOXL2 (−777/−789) site did not affect FOXL2-overexpression SMAD4 promoter activity, indicating that the −1506/+89 region of SMAD4 did not possess the FOXL2 site (Figure 6D).

### 2.6. EMSA for the Confirmation of the Functional Binding of PPARγ, STAT3 and FOXL2 on the TSPO and SMAD4 Promoters

**Figure 7:**
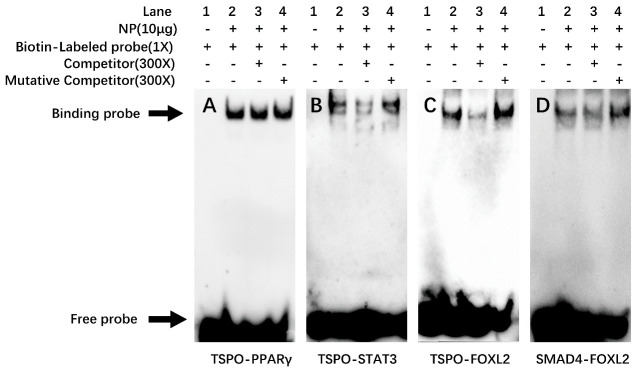
EMSA analysis of predicted SREs. (**A**) −734/−748 binding site of TSPO (TSPO-PPARγ); (**B**) −1507/−1516 binding site of TSPO (TSPO-STAT3); (**C**) −1650/−1663 binding site of TSPO (TSPO- FOXL2); (**D**) −777/−789 binding site of SAMD4 (SAMD4- FOXL2).

EMSA was performed to demonstrate whether these putative binding sites could interact with PPARγ, STAT3, and FOXL2. For the TSPO promoter, the 300-fold unlabeled PPARγ binding sites (−734 bp/−748 bp) did not compete with the PPARγ binding sequence as the probe, indicating that PPARγ could not bind with this region (Figure 7A). However, the 300-fold unlabelled STAT3 and FOXL2 binding sites competed for the binding with the STAT3 and FOXL2 binding sequences as the probe, respectively, and reduced the brightness of the labeled probe, and the 300-fold unlabeled mutated STAT3 and mutated FOXL2 binding region declined this competition, implying that TSPO could be bound by STAT3 (Figure 7B) and FOXL2 (Figure 7C).

For SMAD4 promoter, when the FOXL2 binding sequence was used as the probe, the 300-fold unlabeled FOXL2 binding site (−777 bp/−789 bp) did not compete for the binding, demonstrating that FOXL2 could not bind to this region (Figure 7D).

## 3. Discussion

TSPO and SMAD4 play an important role in steroid synthesis [1,3], but the current research on these two genes mainly focuses on mitochondrial function [14], oxidative stress [15] and signal transduction [16]. Several studies reported that TSPO ligand promoted pregnenolone synthesis [14], and TGF-β/SMAD4 signaling pathway regulated steroid production and ovarian development [17], but these studies are rare in fish. Considering that the characterization of gene sequence is helpful to study their function, we identified the cDNA sequences of TSPO and SMAD4 and explored their mRNA tissue expression from yellow catfish. We also characterized the TSPO and SMAD4 promoters. Our research laid a foundation for further investigation into their function.

Our study found that the protein sequences of *P. fulvidraco* TSPO had similar domains with mammals, such as five α-helix transmembrane structure (TM1–5), the conserved CRAC domain in the C-terminus, in agreement with other studies [18,19]. These indicated that the TSPO was highly evolutionarily conserved, as reported by Jaremko et al. [20]. Our study also demonstrated that the SMAD4 protein possessed six domains, such as N-terminal MH1 domain, C-terminal MH1 domain, SAD domain, DNA Binding motif, NLS domain and NES domain, similar to several studies [21,22]. Studies suggested that these domains were essential for their effector functions responsible for active nucleocytoplasmic shuttling of SMAD4 [23,24]. 

In this study, we demonstrated the tissue distribution of these genes, which provided the basis for elucidating their functions. Our results indicated that their mRNAs were existent in ten tissues in the *P. fulvidraco*, indicating that steroid synthetase plays a wide role in these tissues. Similarly, studies suggested the major sites of steroidogenesis included several gonadal and non-gonadal tissues, such as head kidney, intestine, liver and adipose tissue [25,26]. Our study indicated that TSPO mRNA levels were the highest in the spleen, followed by the testis, kidney and muscle, while there was no significant difference in heart, liver, brain, fat, intestine and ovary. In zebrafish, Rampon et al. [27] reported that TSPO mRNA expression was the highest in the heart, followed by the spinal cord, muscle, ovary, testis and intestine, the lowest in the brain, indicating species-specific differences. In addition, our study indicated that SMAD4 mRNA amounts were predominant in the testis, followed by the liver and ovary, and the lowest in the kidney. In gooses, suggested that SMAD4 mRNAs were expressed in the ovary, hypothalamus and pituitary [28]. The high expression of SAMD4 in the ovary may be related to the secretion of sex hormones. SAMD4, as a transcription factor, can regulate the synthesis of FSH, thus affecting the development of gonads [29]. Their distinctive tissue distribution in *P. fulvidraco* revealed the functional differentiation of these proteins and reflected the tissue-specific metabolic regulation. 

In eukaryotes, the identification of the core regions of promoters is crucial for exploring the mechanism of transcriptional initiation [30]. In the present study, we found Sp1 binding sites in the core region of TSPO promoter. Studies suggested that SP transcriptional sites are important for the basal activity of TSPO promoters [31]. Batarseh et al. [6] found several GC-rich sequences in the proximal region of the TSPO promoter in mouse, in agreement with the present study. Wierstra [32] reported that SP1 directly bound with GC-rich domains and modulated transcription after various stimuli. Thus, we speculated that SP1-rich and GC-rich regions positively regulated TSPO promoter activity. However, we found that this region of SMAD4 promoter had no typical promoter characteristics, such as the high CG content or TATA-box, but had some TATA-like structures (TATAAT) and other potential binding sites, as in other reports [33]. Studies have shown that the promoter lacking TATA possessed various Sp1 binding sites [34]. We also found three Sp1 sites and a downstream core element (DCE) in the core SMAD4 promoter region. These transcription factors ensure the normal initiation of gene transcription [35].

The identification of transcription factor binding sites (TFBS) is significant to reveal the regulatory mechanism of genes [36]. Our study found some binding sites, such as SOX, SRY, GATA, PPARγ, STAT1, Pu.1, AP1, FOXL2 and STAT3 in the promoter region of TSPO, which are similar to other reports in mice [37], reflecting that TSPO participated in many physiological progresses. Rashid et al. [10] pointed out that TSPO was transcriptionally regulated by these transcription factors such as Sp1, Pu.1 and AP1. For SMAD4 promoter, we found Ap1, HNF4α, ZFP and FOXL2 in the SMAD4 promoter region. However, different from human, there are other transcription factors such as F2F, Pit-1 and AP1 in SMAD4 promoters [9]. These indicated that the transcriptional regulation of SMAD4 was complex because maybe multiple transcription factors mediated its regulation, thus playing an important role in fish.

PPARγ, STAT3 and FOXL2 regulate the transcription of key enzymes in steroidogenesis [13,38]. The present study found that PPARγ significantly increased the transcriptional activity of TSPO promoter, while STAT3 and FOXL2 decreased TSPO transcription. These results indicated that PPARγ, STAT3 and FOXL2 differentially regulated steroidogenesis by targeting the TSPO in yellow catfish. However, we identified a functional binding site of STAT3 and FOXL2 in this region, but not PPARγ, after the further site mutagenesis and EMSA. Similarly, Batarseh et al. [39] reported that there was a STAT3 binding site in the TSPO promoter. For the SMAD4 promoter, although FOXL2 overexpression affected the promoter activity, site mutagenesis and EMSA identified SMAD4 is not the target gene of FOXL2. Both SMAD4 and FOXL2 are transcription factors of CYP19A1 [3,38], but FOXL2 didn’t regulate SMAD4. Thus, it is possible that the SMAD4 promoter has other steroidal synthesis regulatory factors.

In summary, we cloned the cDNA sequences of TSPO and SMAD4 genes from yellow catfish, explored their mRNA tissue distribution, and characterized the TSPO and SMAD4 promoter regions. The promoters of TSPO and SMAD4 genes presented different structures on their core regions. The present study demonstrated that TSPO was the direct target gene of STAT3 and FOXL2. These studies are of great significance to demonstrate transcriptional regulation of key genes of steroid synthase in vertebrates.

## 4. Materials and Methods

### 4.1. Ethical Statement and Experimental Procedures

All animal experiments followed the Institutional Ethical Guidelines of Huazhong Agricultural University (HZAU) for using the vertebrates and were approved by the Experimentation Ethics Committee of our university (Wuhan, Hubei, China) (ID Code: Fish-2018-0827, Date: 27 August 2018).

The present study included two experiments. We cloned TSPO and SMAD4 genes, and explored their mRNA tissue distribution in Expt. 1, characterized the upstream 5′ flanking regions of the TSPO and SMAD4 genes and explored their promoter activity by the deletion and mutation analysis in Expt. 2. 

### 4.2. Experimental Animals and Reagents

Juvenile yellow catfish (22.5 ± 3.1 g, mean ± SEM) for cDNA and promoter cloning were purchased from a commercial farm (Wuhan, China). They were maintained in indoor cylindrical fiberglass tanks (300-L water volume) at ambient temperature for 2-week acclimation. All fish were fed a commercial pellet diet twice a day and provided with continuous aeration to maintain the dissolved oxygen level near saturation. At the end of 2-week acclimation, fish were fasted for 24 h and then euthanized with MS-222 (100 mg/L). The heart, brain, liver, kidney, muscle, spleen, fat, intestine, testis and ovary were sampled and stored at −80 °C for the subsequent analysis. The HEK293T cell line was from Cell Resource Center in Fishery College, Huazhong Agricultural University. Dulbecco’s Modified Eagle’s medium (DMEM), fetal bovine serum (FBS) and 0.25% trypsin-EDTA were from Gibco (Waltham, MA, USA). Other reagents were from Sigma-Aldrich (Saint Louis, MO, USA). Using ClonExpress II One Step Cloning Kit (Vazyme, Piscataway, NJ, USA), the overexpression plasmid was labeled with pcDNA3.1 (+) vector.

### 4.3. Expt. 1: Cloning and Tissue Distribution of Gene Expression

#### 4.3.1. RNA Isolation, cDNA Cloning and Sequence Editing

RNA isolation and cDNA cloning of TSPO and SMAD4 followed the protocol described in our previous study [40]. The 3′- and 5′-end sequences were obtained through the nested 3′ and 5′ RACE PCR via the SMART RACE cDNA Amplification Kit (Clontech, USA). The primers were shown in Table S1. The full-length cDNA sequences of TSPO and SMAD4 were edited by EDITSEQ (DNA star) to find the open reading frame (ORF). They were then translated into the amino acid sequence by standard genetic code. Cluster-w multiple alignment algorithm was used to evaluate the sequence alignment and amino acid conservation. On March 5, 2019, NCBI’s online CDD tool (http://www.ncbi.nlm.nih.gov/Structure/cdd/wrpsb.cgi (accessed on 20 April 2021)) was used to analyze the domains. 

#### 4.3.2. Real-Time Quantitative PCR (qPCR)

qPCR method [41] was used to determine the mRNA expression. The primer sequences of genes for qPCR analysis are given in Table S2. The ten housekeeping genes (β-actin, 18s rrna, rpl7, gapdh, hprt, tbp, elfa, tuba and ubce) were selected to analyze their transcriptional stability. The relative expression of genes was calculated via the 2^−ΔΔCt^ method after normalizing to the geometric mean after the best combination of two genes, based on geNorm analysis. Prior to the analysis, we performed experiments to check the stability of housekeeping genes, and β-actin and ubce (M = 1.2669) presented the most stable expression level for analyzing the tissue distribution.

### 4.4. Expt. 2: Structure and Functional Analysis of Promoter 

#### 4.4.1. Cloning of Promoter and Plasmid Construction

Via RNA ligase-mediated rapid amplification of 5′ cDNA ends (RLM-5′ RACE) method, we identified the 5′ cDNA sequences and the transcription start sites (TSS) of TSPO and SMAD4. We cloned the promoter sequence, based on the published draft genome of yellow catfish [42] and our Expt. 1 above, and the protocols were similar to those in our recent studies [43]. Genomic DNA was extracted from yellow catfish tail fins using the commercial kit (Omega, USA). In order to amplify the TSPO and SMAD4 promoter sequences, we designed the specific primers with (Sac I and Hind III) restriction sites (Table S3). To generate the luciferase reporter constructs, we purified the PCR product and pGl3-Basic vectors (Promega, USA) and digested them with corresponding endonucleases, and then these products were ligated via ClonExpress II One Step Cloning Kit (Vazyme, Piscataway, NJ, USA). According to the distance from their TSS, we named the plasmids as pGl3 −2015/+205 of TSPO vector and pGl3 −1506/+89 of SMAD4 vector, respectively. Using the template of pGl3 −2015/+205 of TSPO vector, we produced the plasmids pGl3 −1558/+205, pGl3 −1076/+205 and pGl3 −504/+205 of TSPO vector. Similarly, using pGl3 −1506/+89 of SMAD4 vector as a template, we generated plasmids pGl3 −999/+89 and pGl3 −559/+89.

#### 4.4.2. Sequence Analysis

For sequences analysis of the TSPO and SMAD4 promoters in yellow catfish, their TFBSs were predicted by the online tools on August 9, 2020 (http://www.genomatix.de/ and http://jaspar.genereg.net/ (accessed on 20 April 2021)). We used the Clustal-W multiple alignment to evaluate sequence alignments.

#### 4.4.3. Transfections and Luciferase Assays

We transiently transfected the plasmid into HEK293T cells with Lipofectamine 2000 (Invitrogen, Carlsbad, CA, USA), based on the manufacturer’s instruction. The reporter plasmids were used in equimolar amounts in Opti-MEM (Invitrogen, Carlsbad, CA, USA). They were co-transfected with 20 ng pRL-TK as a control. After 4-h transfection, the medium was replaced by DMEM plus 10% FBS. Then, after 24-h incubation, we harvested cells to determine the luciferase activity by Dual-Luciferase Reporter Assay System (Promega, Madison, WI, USA) after calculating the ratio of firefly luciferase to Renilla luciferase. These experiments were performed in triplicates.

To explore the overexpression-induced changes of promoter activities, we co-transfected the overexpression plasmid or the same amount of pcDNA3.1 (+) plasmid (control) with the TSPO and SMAD4 luciferase reporter plasmids into HEK293T cells using Lipofectamine 2000 (Invitrogen, Carlsbad, CA, USA). The remaining steps are the same as above.

#### 4.4.4. Site-Mutation Analysis of Binding Sites on the TSPO and SMAD4 Promoters

To identify the corresponding PPARγ, STAT3 and FOXL2 binding sites on the yellow catfish TSPO and SMAD4 promoters, we performed the site-directed mutagenesis, based on the instruction of QuickChange II Site-Directed Mutagenesis Kit (Vazyme, Piscataway, NJ, USA). The pGl3-TSPO-2015 and pGl3-SMAD4-1558 were used as the template, respectively. The mutagenesis primers were presented in Table S3. These mutant constructs were named as Mutation-PPARγ, Mutation-STAT3 and Mutation-FOXL2, respectively. To study the overexpression-induced changes of PPARγ, STAT3 and FOXL2 binding sites, we co-transfected overexpression plasmid (300 ng) or pcDNA3.1 (+) plasmid (control, 300 ng) with wild-type plasmid (500 ng) or mutation plasmid (500 ng) into HEK293T cells by Lipofectamine 2000 (Invitrogen, Carlsbad, CA, USA). The remaining steps are the same as above.

#### 4.4.5. Electrophoretic Mobility-Shift Assay (EMSA)

EMSA was performed to analyze the PPARγ, STAT3 and FOXL2 functional binding sites on the regions of TSPO and SMAD4 promoters after Xu et al. [43]. Cytoplasmic and nuclear extracts were obtained with the same protocols of Xu et al. [43]. We extracted the nuclear proteins from HEK293T cells, and used the bicinchoninic acid assay (BCA) to determine the protein content. Table S4 listed the oligonucleotide sequences for EMSA.

### 4.5. Statistical Analysis

The data were showed as means ± standard error of mean (SEM). Before the statistical analysis, all data were tested for distribution normality using the Kolmogornov-Smirnov test. Using the unpaired two-tailed Student’s *t* test, we determined the differences between two groups. Difference was thought to be statistically significant at *p* < 0.05. The SPSS 19.0 for Windows (SPSS Chicago, IL, USA) was used for the statistical analyses.

## Figures and Tables

**Table 1:** The sequence information of TSPO and SMAD4 Genes from *P. fulvidraco.*

Gene	Accession No.	5′ UTR (bp)	ORF (bp)	3′ UTR (bp)	Full Length (bp)	Protein (aa)
TSPO	MN188059	116	486	350	952	161
SMAD4	MN188058	202	1725	294	2221	574

**Table 2:** Amino acid sequence identity of TSPO and SMAD4 Genes between *P. fulvidraco* and other species (%).

Gene	*Ictalurus punctatus*	*Danio rerio*	*Xenopus tropicalis*	*Homo sapiens*	*Rattus norvegicus*
TSPO	69.12	61.29	50.69	41.94	39.63
SMAD4	94.19	63.93	65.64	66.32	66.32

Notes: Accession numbers as follows (the order is *Ictalurus punctatus*, *Danio rerio*, *Xenopus tropicalis*, *Homo sapiens* and *Rattus norvegicus*): TSPO (XM 017479065.1, NM 001006032.2, XM 012956428.2, NM 001256531.1, NM 012515.2; SMAD4 (XM 017471854.1, EU489481.1, XM 002934439.4, NM 005359.5, NM 019275.3).

## Data Availability

Data is contained within the article or Supplementary Materials.

## Supplementary Materials

The following are available online at https://www.mdpi.com/article/10.3390/ijms22094505/s1, Table S1: Nucleotide sequences of the primers used for the cDNA cloning from *P. fulvidraco*, Table S2: Primers used for real-time quantitative PCR analysis, Table S3: Primers used for cloning and functional analysis of promoters in the experiments, Table S4: Primers used for electrophoretic mobility-shift assay. Figure S1: Multiple amino acid sequence alignment of TSPO from *P. fulvidraco* and other species, Figure S2: Multiple amino acid sequence alignment of SMAD4 from *P. fulvidraco* and other species.

## Abbreviations

TSPO, translocator protein; STAR, steroidogenic acute regulatory protein; SMAD4, mothers against decapentaplegic homolog 4; SAD, SMAD4 activation domain; NLS, nuclear localization signal; NES, nuclear export signal; DMEM, Dulbecco’s Modified Eagles Medium; FBS, fetal bovine serum; PBS, phosphate-buffer saline; TFBS, transcriptional factor binding sites; TSS, transcription start site; Sp, specificity protein; AP1, activator protein-1; HNF4α, hepatic nuclear factor 4α; PPARγ, peroxisome proliferator-activated receptor γ; Pu.1, PU box binding-1; STAT, signal transducer and activator of transcription; FOXL2, forkhead box l2; SOX1, SRY-box transcription factor 1; SRY, sex determining region Y
