# Supplementary material for: Molecular Characterization and Functional Analysis of Two Steroidogenic Genes TSPO and SMAD4 in Yellow Catfish
Source: Int J Mol Sci. 2021 Apr 26;22(9):4505. doi: 10.3390/ijms22094505 (PMC8123483; doi:10.3390/ijms22094505)
Supplement: Supplementary file 1 [file ijms-22-04505-s001.zip › ijms-1178529-supplementary.pdf]

Table S1. Nucleotide sequences of the primers used for the cDNA cloning from *P. fulvidraco*.

Primers	Sequences (5'-3')
**Primers for 3'-RACE PCR**	
3’GS-TSPO-O	CGCAATGCCACTCTTCTC
3’GS-TSPO-I	CTCAACTACTGTATCTGGAGGGA
3’GS-SMAD4-O	GCACAAAATCTACCCAGGAG
3’GS-SMAD4-I	GGATTACCCACGCCAAAG
3’GS-RACE Outer	TACCGTCGTTCCACTAGTGATTT
3’GS- RACE Inner	CGCGGATCCTCCACTAGTGATTTCACTATAGG
**Primers for 5'-RACE PCR**	
5’GS-TSPO-O	CGGTTTGTTAAGTGAGGC
5’GS-TSPO-I	GCTACCAAAGATCCCACC
5’GS-SMAD4-O	GAATCCAGCTCATCCTTCT
5’GS-SMAD4-I	GCTTGGCAAATCCCTCAT
5' RACE Outer	CATGGCTACATGCTGACAGCCTA
5' RACE Inner	CGCGGATCCACAGCCTACTGATGATCAGTCGATG

Notes: K-G/T; M-A/C; R-A/G; S-G/T; Y-C/T; B-G/T/C; D-G/A/T; H-A/T/C; V-G/A/C; N-A/T/G/C.

Table S2. Primers used for real-time quantitative PCR analysis

Genes	Forward primer (5’-3’)	Reverse primer (5’-3’)	Size bp	Accession No.
**TSPO**	CAGAAGATGCTGTGGTCCCA	AGCAAAGCCAAGCCAGGTAT	213	MN188059
**SMAD4**	CACAGCCAGTCTCCAACCAT	CATCTCCTTCCCCACGACAC	258	MN188058
**β-actin**	GCACAGTAAAGGCGTTGTGA	ACATCTGCTGGAAGGTGGAC	136	EU161066
**18S-rRNA**	AGCTCGTAGTTGGATCTCGG	CGGGTATTCAGGCGAGTTTG	196	KP938527
**RPL7**	GGCAAATGTACAGGAGCGAG	GCCTTGTTGAGCTTGACGAA	199	KP938522
**GAPDH**	TTTCAGCGAGAGAGACCCAG	ATGACTCTCTTGGCACCTCC	132	KP938521
**HPRT**	ATGCTTCTGACCTGGAACGT	TTGCGGTTCAGTGCTTTGAT	181	KP938523
**TBP**	AGCAAAGAGTGAGGAGCAGT	TAGGGGTAGTCGATGGGGAA	150	KU886307
**ELFA**	GTCTGGAGATGCTGCCATTG	AGCCTTCTTCTCAACGCTCT	151	KP938526
**TUBA**	TCAAAGCTGGAGTTCTCGGT	AATGGCCTCGTTATCCACCA	135	KP938526
**UBCE**	TCAAGAAGAGCCAGTGGAGG	TAGGGGTAGTCGATGGGGAA	150	KP938524

Abbreviations: ELFA, translation elongation factor; GAPDH, Glyceraldehyde-3-phosphate dehydrogenase; HPRT, hypoxanthine-guanine phosphoribosyltransferase; RPL7, ribosomal protein L7; TBP, TATA-box-binding protein; TUBA, tubulin alpha chain; UBCE, ubiquitin-conjugating enzyme.

Table S3. Primers used for cloning and functional analysis of promoters in the experiments

Name	Primer	Sequence(5'-3')
**Primers for promoter construct**		
TSPO	RT-PCR R	GGCTTGGTTATGAAAGAAGGAC
	RT-PCR F	CTTAGCAGACAGAGTGGGAATG
	pGl3-504/+205 F	ctatcgataggtaccgagctcCTATCTGCAACCCTTCTCTTCATTC
	pGl3-1076/+205 F	ctatcgataggtaccgagctcCTTTAAATCTGGGAATTCAGTATAACCT
	pGl3-1558/+205 F	ctatcgataggtaccgagctcTACAATCATGTTAGTCTGAGGACATTAGG
	pGl3-2015/+205 F	ctatcgataggtaccgagctcCTTAGCAGACAGAGTGGGAATGG
	pGl3-2015/+205 R	cagtaccggaatgccaagcttGGCTTGGTTATGAAAGAAGGACG
SMAD4	RT-PCR R	GGATGTGCGAATGAGGG
	RT-PCR F	CCTTCCACCTGGTCTCC
	pGl3-559/+89 F	ctatcgataggtaccgagctcTTGGACTTTTTACATTTGAATTTAAAAG
	pGl3-999/+89 F	ctatcgataggtaccgagctcAATCAAATCAAATCAAATCAAATTTTATT
	pGl3-1506/+89 F	ctatcgataggtaccgagctcAGTTAGAGATATAGGGTGTATCTGAAGATAGA
	pGl3-1506/+89 R	cagtaccggaatgccaagcttCCCTCATTCGCACATCCTGC
**Primers for deleting PPARγ, STAT3 and FOXL2 binding sites of promoters**		
TSPO-Mutation	-PPARγ F	gggattgaagttaaagtttCTTTTTGTCCTAATGTAGCTCCCA
	-PPARγ R	aactttaacttcaatccctTTATCTTATTTGGCACATCATTTGG
	-STAT3 F	agcagggaaccccccatggaTTAATGGAACAATTTTTTTAAATCACTG
	-STAT3 R	atggggggttccctgctcTGCTGTAAATACATTCTGGAAGCAT
	-FOXL2 F	attcgcctcccacgtcacAATAACTGATTTCATTATATTTGGGTGAT
	-FOXL3 R	tgacgtgggaggcgaatgCACATTTGTTTGCAAGTAATTGGTT
SMAD4-Mutation	-FOXL2 F	aaccgcgacctcgcgcgatGAAGAAATAATGTATATACATCTGCATAATATG
	-FOXL3 R	tcgcgcgaggtcgcggtttATATAGTTTTTCTTATATAGTTTTCTTATACAGTT TATACT
**Primers for overexpression plasmids of PPARγ, STAT3 and FOXL2 binding sites into pcDNA3.1 (+) vector**		
PPARγ	F	ctagcgtttaaacttaagcttATGGTGGACACACAGACGTTTTC
	R	aacgggccctctagactcgagCTACTTATCGTCGTCATCCTTGTAATC
STAT3	F	ctagcgtttaaacttaagcttATGGCCCAGTGGAATCAGTTG
	R	aacgggccctctagactcgagTCACTTATCGTCGTCATCCTTGTAA
FOXL2	F	ctagcgtttaaacttaagcttATGATGGCCTCCTACTTGGGC
	R	aacgggccctctagactcgagTTACTTATCGTCGTCATCCTTGTAATC

Table S4. Primers used for electrophoretic mobility-shift assay

Primers		Forward primer (5'-3')	Reverse primer (5'-3')
TSPO-PPARγ	Biotin-probe	Biotin-TGCCACCTGGCCCAG	Biotin-CTGGGCCAGGTGGCA
	Mutative-competitor	GATTGAAGTTAAAGT	ACTTTAACTTCAATC
TSPO-STAT3	Biotin-probe	Biotin-TTTCCAAAAAA	Biotin-TTTTTTGGAAA
	Mutative-competitor	GGGAACCCCCC	GGGGGGTTCCC
TSPO-FOXL2	Biotin-probe	Biotin-GATAAGTAAATATG	Biotin-CATATTTACTTATC
	Mutative-competitor	TCGCCTCCCACGT	ACGTGGGAGGCGA
SMAD4-FOXL2	Biotin-probe	Biotin-AATATGAAAATATA	Biotin-TATATTTTCATATT
	Mutative-competitor	CCGCGACCTCGCGC	GCGCGAGGTCGCGG

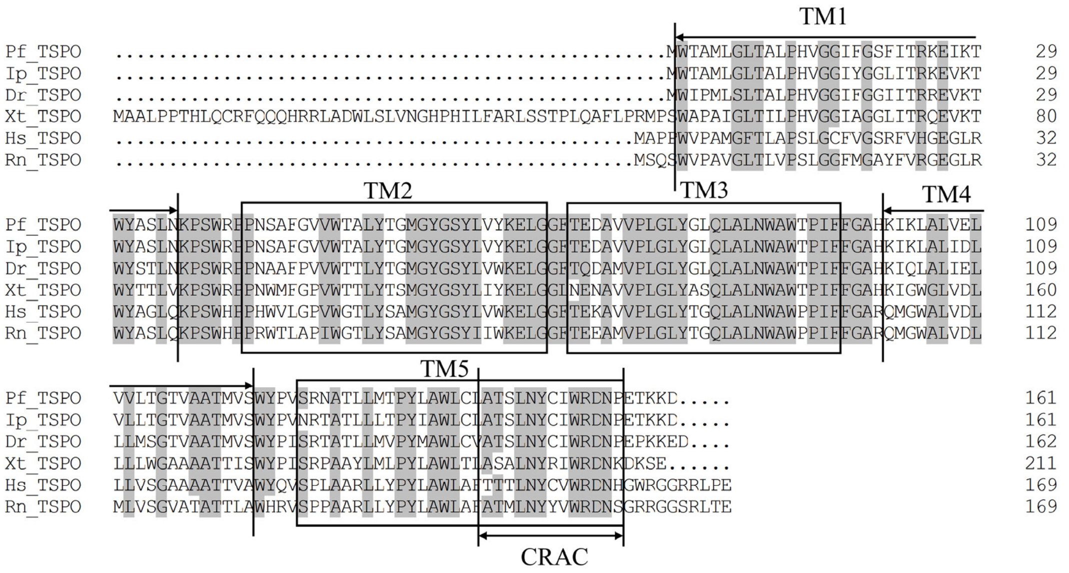

Figure S1. Multiple amino acid sequence alignment of *TSPO* from *P. fulvidraco* and other species. Accession numbers as follows (the order is *Pelteobagrus fulvidraco* (Pf), *Ictalurus punctatus* (Ip), *Danio rerio* (Dr), *Xenopus tropicalis* (Xt), *Homo sapiens* (Hs) and *Rattus norvegicus* (Rn)): XM_017479065.1, NM_001006032.2, XM_012956428.2.1, NM_001256531.1, NM_012515.2). The arrows above and below the sequence indicate the helix transmembrane structure (TM1-5) and the CRAC domain, respectively.

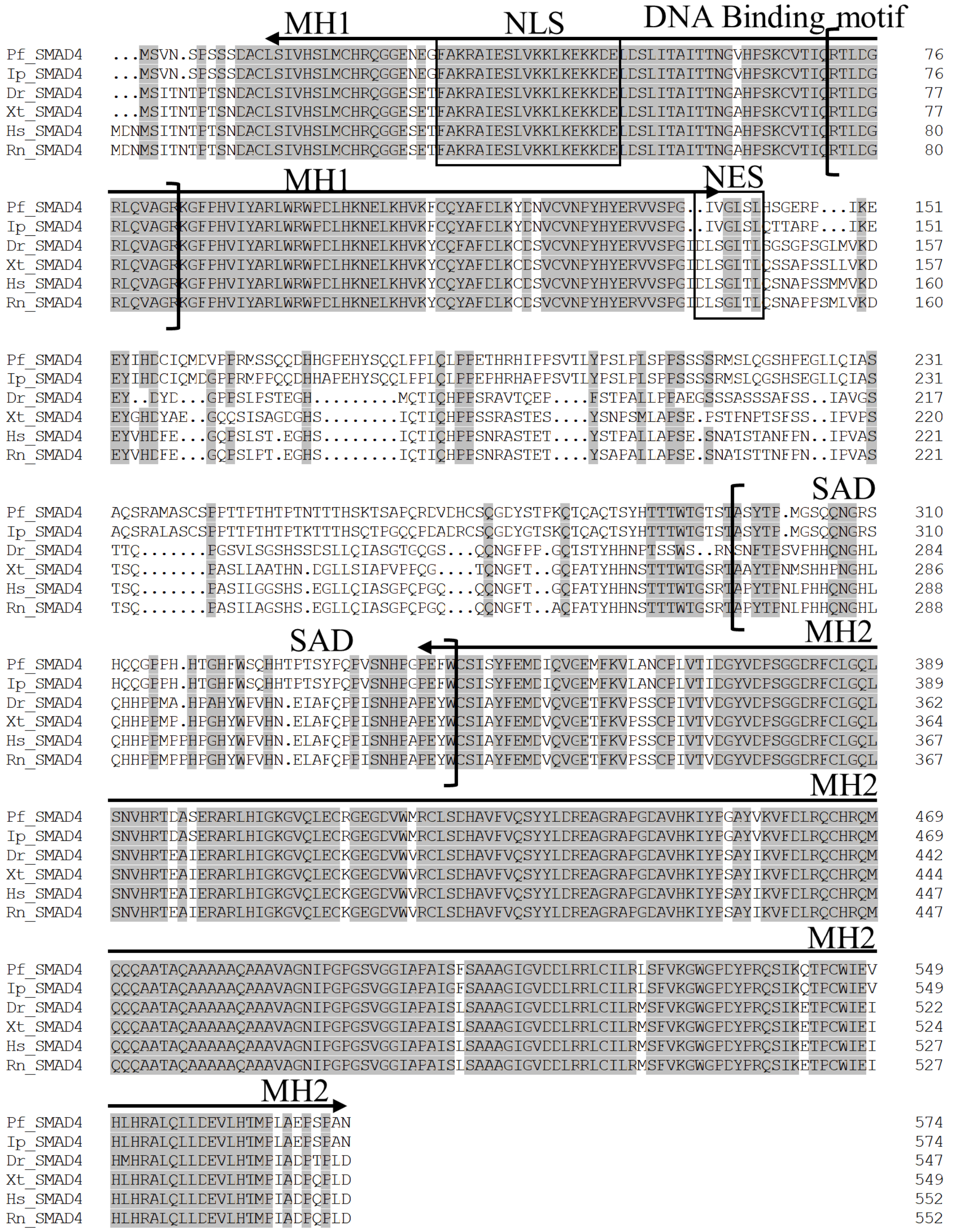

Figure S2. Multiple amino acid sequence alignment of *SMAD4* from *P. fulvidraco* and other species. Accession numbers as follows (the order is *Pelteobagrus fulvidraco* (Pf), *Ictalurus punctatus* (Ip), *Danio rerio* (Dr), *Xenopus tropicalis* (Xt*)*, *Homo sapiens* (Hs) and *Rattus norvegicus* (Rn)): XM_017471854.1, EU489481.1, XM_002934439.4, NM_005359.5, NM_019275.3). Arrow below the sequences represent the N-terminal MH1 and the C-terminal MH2. The other conserved domain (nuclear localization signal (NLS) domain, nuclear export signal (NES) domain) are boxed. The DNA Binding motif and SAD domain are represented by parentheses.
